# A generalized quantitative interpretation of dark-field contrast for highly concentrated microsphere suspensions

**DOI:** 10.1038/srep35259

**Published:** 2016-10-13

**Authors:** Spyridon Gkoumas, Pablo Villanueva-Perez, Zhentian Wang, Lucia Romano, Matteo Abis, Marco Stampanoni

**Affiliations:** 1 Swiss Light Source, Paul Scherrer Institute, 5232 Villigen, Switzerland; 2 Institute for Biomedical Engineering University and ETH Zürich, 8092 Zürich, Switzerland; 3 Department of Physics and IMM-CNR, University of Catania, 64 via S. Sofia, I-95123 Catania, Italy

## Abstract

In X-ray grating interferometry, dark-field contrast arises due to partial extinction of the detected interference fringes. This is also called visibility reduction and is attributed to small-angle scattering from unresolved structures in the imaged object. In recent years, analytical quantitative frameworks of dark-field contrast have been developed for highly diluted monodisperse microsphere suspensions with maximum 6% volume fraction. These frameworks assume that scattering particles are separated by large enough distances, which make any interparticle scattering interference negligible. In this paper, we start from the small-angle scattering intensity equation and, by linking Fourier and real-space, we introduce the structure factor and thus extend the analytical and experimental quantitative interpretation of dark-field contrast, for a range of suspensions with volume fractions reaching 40%. The structure factor accounts for interparticle scattering interference. Without introducing any additional fitting parameters, we successfully predict the experimental values measured at the TOMCAT beamline, Swiss Light Source. Finally, we apply this theoretical framework to an experiment probing a range of system correlation lengths by acquiring dark-field images at different energies. This proposed method has the potential to be applied in single-shot-mode using a polychromatic X-ray tube setup and a single-photon-counting energy-resolving detector.

X-ray grating interferometry (XGI) is a powerful imaging technique originally developed for synchrotron radiation using a Talbot arrangement^1^,^2^,^3^ and later transferred to polychromatic X-ray tube setups through a Talbot-Lau configuration^4^. Through the implementation of large area gratings, XGI can provide full-field images of attenuation (absorption), refraction (differential-phase) and small-angle scattering (dark-field) contrasts simultaneously.

Dark-field (*S*) provides contrast from unresolvable microstructures within an imaged object. It is often stated that it is related to ultra-small-angle X-ray scattering (USAXS)^5^ and it is defined as


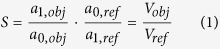


where *a*_*i*_ is the magnitude of the i-th Fourier component of the phase-stepping curve and *V* = 2*a*_1_/*a*_0_ is defined as the visibility^6^.

XGI paved the way for the recent broad and extensive research on possible applications of dark-field contrast imaging and this yielded numerous studies on biological samples such as bone^7^,^8^,^9^, teeth^10^, breast tissue^11^,^12^,^13^,^14^, pre-clinical lung cases^15^,^16^ and kidney stone differentiation^17^. In addition to research towards biological dark-field imaging, several groups developed analytical theoretical frameworks for the quantification of dark-field, working through either wave optics propagation^5^,^18^ or analysis equivalent to spin-echo small-angle neutron scattering^19^. Although simulations are outside the scope of the work presented in this article, in recent years several groups have presented results from simulation frameworks towards the interpretation and quantification of dark-field signal^20^,^21^,^22^,^23^. Among these, Ritter *et al*. use one sample of dry microspheres in order to obtain experimental validation for their simulation framework^21^.

The existing analytical theoretical approaches have been validated experimentally by using highly diluted monodisperse microsphere suspensions. The volume fraction for such control samples was deliberately kept below the 6% level, to justify the assumption of spheres or particles being separated by large enough distances to make any interparticle scattering interference negligible^5^,^18^,^19^. On the other hand, to potentially extract quantitative dark-field information from dense biological or material science samples, it is necessary to study both the theoretical and experimental dependence of dark-field contrast for highly concentrated systems (volume fractions much higher than 6%). In the Fourier domain, this extension is equivalent to introducing interparticle scattering interference in the form of a structure factor and was reported already by Zernike and Prins in 1927^24^.

In this paper, we start by analytically calculating the well-established USAXS intensity equation using as input the known experimental parameters of the XGI setup, as well as the physical specifications of the samples used. By performing Fourier analysis and making the transition from momentum to real-space, we assume highly diluted microsphere suspensions and thus arrive to the exact same prediction as proposed by wave-propagation^5^,^18^ and spin-echo small-angle neutron scattering theory models^19^. Through this direct calculation we verify the undisputed link between dark-field and USAXS, as also shown for neutrons by M. Strobl^19^. Furthermore, we extend the current analytical interpretation of dark-field by implementing the structure factor for an adhesive hard sphere potential, as proposed by Baxter^25^.

Without introducing any additional fitting parameters we present a theoretical and experimental extension of quantitative dark-field imaging, using monodisperse microsphere suspensions for a range of volume fractions reaching 40%. Finally, we propose an alternative method for probing a certain range of system correlation lengths or scattering vectors. We achieve this experimentally by performing dark-field imaging at different energies, while using the XGI system with fixed distances between system components and sample. By applying the developed theoretical framework, we illustrate the potential of retrieving information from highly dense samples composed by microstructures of unresolved sizes. With the use of a single-photon-counting energy-resolving detector, this method has the future potential to be transferred to polychromatic X-ray tube setups^26^. More importantly, for a narrow and well-defined scattering vector range it can emulate the USAXS process while exploiting the large field of view of the imaging regime.

## Results

### The link between dark-field and USAXS

For a given sample and scattering vector *q*, the differential scattering cross-section 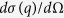 or scattering intensity *I*(*q*) can be physically described as the number of scattered photons per unit time, unit solid angle and unit volume of the sample, relative to the incident photon flux^27^.

For monodisperse spherical particles in a homogeneous solvent, the intensity of the scattered radiation can be expressed as


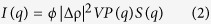


where *ϕ* is the volume fraction between particles and solvent material and *V* is the volume of the particle. *q* is the magnitude of the scattering vector or momentum transfer and is given by


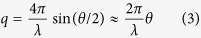


where *λ* is the wavelength and *θ* the scattering angle. Using the small-angle approximation we can estimate that sin(*θ*/2) ≈ *θ*/2. Δ*ρ* is the difference in electron density between the particles and the solvent material and is proportional to Δ*χ*, which is the difference in the complex index of refraction between the particles and the solvent, according to


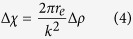


where *k* is the wave vector and *r*_*e*_ the classical electron radius. Δ*χ* can be calculated by using *δ* and *β* tabulated values according to Δ*χ* = Δ*δ* + *j*Δ*β*, where *δ* and *β* are the real and imaginary parts of the refractive index and they both depend on the material’s chemical composition and the X-ray wavelength. *P*(*q*) is the particle form factor describing the structure of a single particle and it satisfies *P*(*q* = 0) = 1. In the case of a homogeneous sphere, the amplitude of the form factor is given by


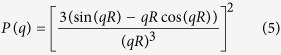


where *R* is the sphere radius. The structure factor *S*(*q*) is related to the interference occurring between scattering particles. For highly diluted systems *S*(*q*) = 1 and thus it can be neglected. On the other hand, for particle systems with higher volume fractions, *S*(*q*) becomes highly influential and varies the scattering intensity via the product *P*(*q*)*S*(*q*).

**Figure 1:**
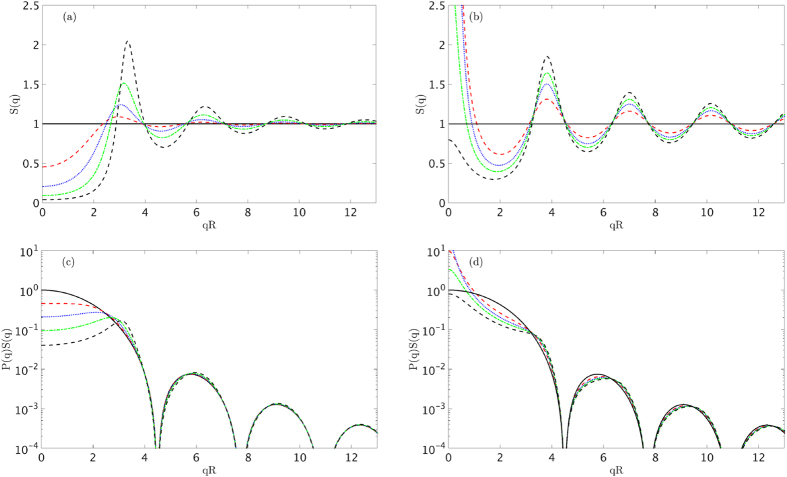
Adhesive hard sphere structure factor dependence on *qR* for minimum (**a**) and maximum (**b**) adhesion, for a number of volume fractions. Influence of the structure factor to the scattering intensity via the product *P*(*q*)*S*(*q*) for minimum (**c**) and maximum (**d**) adhesion. For all graphs the volume fractions range from near 0% (black – solid line), 10% (red – dashed line), 20% (blue – dotted line), 30% (green – dash-dotted line) and 40% (black – dashed line) in 10% steps.

To illustrate this, we implement the analytic expression of the adhesive hard sphere structure factor^25^ for minimum and maximum sphere surface adhesion, with the adhesion parameter *τ* being equal to 99 and 0.1 respectively, using increasing fractions of volume from 0% to 40% in 10% steps as shown in Fig. 1. For negligible minimum adhesion (*τ* = 99), the potential reduces to one of a solely repulsive hard core, known as the hard sphere structure factor^28^, and the interaction of the particles’ core and surface takes place at the closest approach through a very steep, nearly infinite pair potential. As the adhesion increases, the particles are also characterized by a delta function, short-range attractive potential at their surface. This creates a rectangular attractive well type potential of certain width and depth known as the Baxter model for adhesive hard spheres^25^. The maximum adhesion (*τ* = 0.1) was chosen at the limit where Baxter’s model fails. It can be observed in Fig. 1, that the structure factor causes significant perturbation to the single sphere form factor for low *qR* and high fraction of volume values.

XGI is a near field technique. Hence, all XGI dark-field measurements are also performed in the near field regime. The dark-field signal which can be obtained by XGI can be expressed in terms of the autocorrelation function, as also reported by Yashiro *et al*.^5^, Lynch *et al*.^18^ and Strobl^19^. By applying the Fourier transform to that autocorrelation function we can relate it to the far field intensity distribution^5^,^19^. Therefore, there is a direct and unambiguous relation between the autocorrelation in the near field and intensities in the far field.

In this paper, we implement the aforementioned theoretical link in the opposite direction. We begin with equations in the far field and by applying the Fourier transform to the scattering intensity defined by equation (2), we can compute equation (6) which expresses the autocorrelation function and its relation to the dark-field extinction coefficient *μ*_*d*_. Thus, we verify the direct analytical link between the dark-field and USAXS regimes. By introducing the structure factor, we extend the current analytical and quantitative interpretation of dark-field signal for a wide range of diluted to highly concentrated microsphere suspensions. For the latter case of highly concentrated suspensions, and thus high fractions of volume *ϕ*, the structure factor accounts for the influence of interparticle interference on dark-field signal.


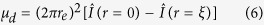


where 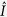 is the Fourier transform of *I*. *ξ* is the XGI system’s correlation length and is given by the expression 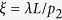. *L* is the inter-grating distance between the phase grating G1 and the analyzer or absorption grating G2 and *p*_2_ is the period of the detected interference fringes when the sample is close and prior to G1 in parallel illumination. Using the small-angle approximation the system’s correlation length can also be expressed as 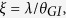 hence defining the XGI characteristic scattering vector 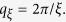 A more general expression of the correlation length for an XGI system is discussed by Donath *et al*.^29^.

### Quantitative dark-field for highly concentrated suspensions

To validate our theoretical approach, we performed XGI experiments using colloidal suspensions of SiO_2_ microspheres in glycerin (C_3_H_8_O_3_) with increasingly higher concentrations. The experiments were carried out at the TOMCAT beamline, Swiss Light Source, at the energy of 25 keV and for that specific configuration the theoretical correlation length was equal to *ξ* = 2.98 *μ*m. The experimentally achieved correlation length including experimental uncertainties was equal to *ξ* = 3.0 ± 0.2 *μ*m. The microsphere diameters used for this experiment were 7.75 and 1.86 *μ*m and the achieved fractions of volume ranged approximately between 5% and 40%. By making the valid assumption that a single type of material was used for each sample of spheres, the experimental values of the dark-field extinction coefficient were calculated according to


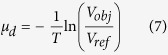


where *T* is the thickness of the sample.

**Figure 2:**
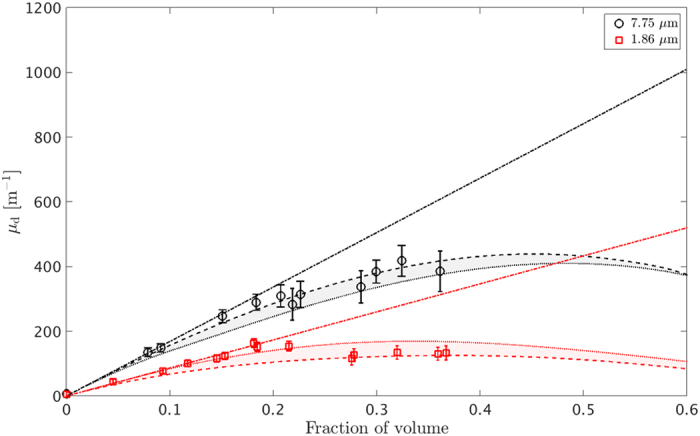
Experimental *μ*_*d*_ values as a function of volume fraction for 7.75 (black circles) and 1.86 *μm* (red squares) diameter microspheres. The dash-dotted, dashed and dotted lines represent theoretical calculations for single sphere, minimum and maximum adhesive hard sphere models, respectively.

Figure 2 shows the experimental dark-field extinction coefficient values as a function of increasing volume fractions for 7.75 and 1.86 *μ*m microspheres. The dot-dashed lines represent the theoretical predictions as proposed by models until now^5^,^18^,^19^, excluding interparticle scattering interference. These models are valid for highly diluted samples (<10% volume fraction) and follow a linear relation between dark-field signal and volume fraction, since each sphere contributes incoherently to the total scattering intensity. In the USAXS regime, this corresponds to a constant *S*(*q*) = 1. The dashed and dotted lines illustrate the theoretical prediction according to our proposed calculation and without any fitting performed for minimum and maximum surface adhesion, respectively. The vertical error bars indicate the standard deviation of the dark-field extinction coefficient calculated using a large region of interest for each imaged suspension. For minimum adhesion, the adhesive hard sphere structure factor essentially reduced to the hard sphere one. The shadow areas between the dash and dotted lines represent the range of possible *μ*_*d*_ values between a non-adhesive and maximum adhesive *S*(*q*).

Our results show that the grating interferometer used with *ξ* = 3 ± 0.2 *μ*m is more sensitive to the 7.75 diameter *μm* spheres compared to the 1.86 *μm* ones. As discussed in the next section, this sensitivity depends both on the system’s correlation length and the microsphere size. It is evident that by introducing a structure factor, the extended theoretical prediction is in good agreement with the experimental results up to volume fractions approaching 40%. If compared to the uncorrelated spheres contribution case, the *S*(*q*) inclusive theoretical curves illustrate a modulated increase of dark-field signal with increasing volume fraction. It is particularly interesting to notice that the latter curves also present a certain maximum point, beyond which dark-field begins to decrease, making it possible to obtain the same dark-field contrast value for two different concentrations. This signifies the necessity of including *S*(*q*) or an equivalent factor when performing quantitative dark-field imaging of biological or material science granular samples, to avoid misinterpreting contrast variations caused by the combined effects of difference in particle size and concentrations.

### Probing a range of *ξ* values through energy selectivity

The variation of the correlation length *ξ* of an XGI can probe different object sizes. Several methods have been reported and can be applied to access a certain *ξ* range. W. Yashiro *et al*.^5^ and S. K. Lynch *et al*.^18^ vary the Talbot order by changing the energy and inter-grating distance 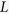 for samples placed prior to and after G1, respectively. M. Strobl^19^ and F. Prade *et al*.^30^ change the position of the sample along the optical z-axis for samples placed after G1.

We propose an alternative method of varying *ξ* based on changing X-ray energy. This enables keeping the XGI components, the Talbot order and the sample position fixed along the optical z-axis. It is important to note that according to equations 2, 4 and 6, *μ*_*d*_ includes additional energy dependent factors other than *ξ*. These are all changed when varying the energy. More specifically, *μ*_*d*_ is proportional to the factor 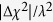 and thus has a quadratic dependence on energy. This is a major difference when calculating the dependence of *μ*_*d*_ as a function of *ξ*, between the proposed method and existing ones^5^,^19^,^30^. Similar to the normalization method described by S. K. Lynch *et al*.^18^, it is possible to decouple the dark-field coefficient *μ*_*d*_ from this energy dependent factor and thus introduce the normalized *μ*_*d*,*norm*_ according to


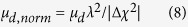


Although the proposed energy change approach does not immediately appear as an attractive option for synchrotron monochromatic experiments, it carries the potential of being exploited in a single phase-stepping acquisition by an X-ray tube table-top setup of fixed magnification and large field of view, with the use of a high-Z single-photon-counting energy-resolving semiconductor detector. Nevertheless, it is a well known fact that XGI has a certain spectral acceptance and visibility varies as a function of energy^26^,^31^. This will cause a variation of the dark-field’s signal to noise ratio probed by our proposed method. Thus, the proposed method has to be selectively applied for energy bins whose visibility reduction is above the system’s noise level.

In this section we present proof of concept experimental results of this method, taken at the TOMCAT beamline, Swiss Light Source. The energies used ranged from 22 up to 37 *keV* in 3 *keV* steps. The samples imaged were 10.0, 6.4 and 2.0 *μm* diameter microspheres in water. To simulate a random granular configuration, the samples were left to reach a sedimentation state with measured volume fractions of 25%, 35% and 50%, respectively. In this way, high fraction volume stability was ensured throughout the energy range measurements.

**Figure 3:**
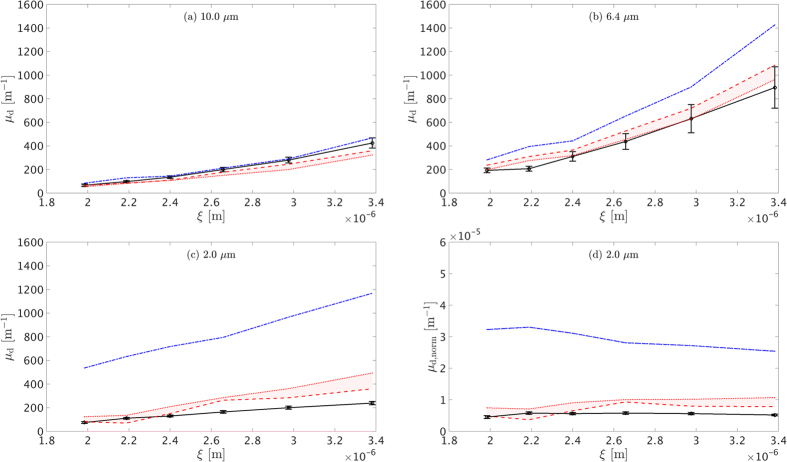
Experimental *μ*_*d*_ values as a function of *ξ* (black circles), by means of altering the X-ray energy for (**a**) 10, (**b**) 6.4 and (**c**) 2.0 *μm* diameter microsphere cases in sedimentation. (**d**) Represents the same data as (**c**) but normalized for the effect of the energy dependent proportionality factor. The blue-dash-dotted, red-dashed and red-dotted lines represent theoretical calculations for single sphere, minimum and maximum adhesive hard sphere models, respectively.

For all the graphs of Fig. 3, our theoretical structure factor inclusive approach is in good agreement with the experimental data. Similarly to Fig. 2, the shadowed red areas indicate the range of possible *μ*_*d*_ values for a minimum and maximum adhesive hard sphere *S*(*q*). Specifically for the 2.0 *μm* diameter case, it is noteworthy that although the volume fraction was equal to 50% and indicates a densely packed system of spheres, the theoretically calculated *S*(*q*) inclusive values still remain in relatively good agreement with the measured ones. In contrast to that, the single sphere theoretical prediction fails to reproduce the measured dark-field signal for the whole *ξ* range presented. Moreover, Fig. 3(c,d) illustrate the difference between *μ*_*d*_ and 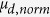 as a function of *ξ*, respectively. The normalized coefficient demonstrates a saturated constant value, while the direct coefficient a characteristic quadratic increase due to the energy dependent proportionality factor 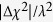.

This difference between the uncorrelated spheres and adhesive hard sphere models appears to become less significant when the ratio between the sphere diameter and the correlation length increases. For our experimental results this effect appears more pronounced for the 10 *μm* spheres, which also exhibit the lowest volume fraction between the three samples. For this case all the models predict similar values and closely match the experimental data. Therefore, in such a scenario the XGI system is not very sensitive to the effects of the structure factor, which asymptotically approaches unity with increasing 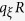 values as shown in Fig. 1(a,b). When such conditions apply, the uncorrelated spheres scattering intensity model can be used as a reasonable approximation to predict experimental values.

This validation between theory and experiment for a range of different *ξ* values and different diameter microspheres proves that it is feasible to map the correlation length successfully through energy selectivity. The impact of such a method lies in the fact that it can be potentially transferred to polychromatic X-ray tube XGI setups and exploit the large field of view via cutting-edge energy-resolving detectors.

**Figure 4:**
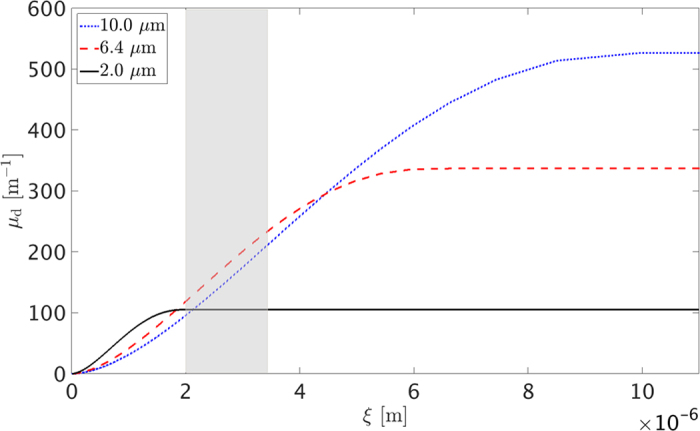
Calculated theoretical curves of *μ*_*d*_ variation as a function of *ξ* for 2 (black – solid line), 6.4 (red – dashed line) and 10 *μm* (blue – dotted line) spheres, respectively. The gray shadow area illustrates the *ξ* range probed by our multiple energy experiments.

Moreover as shown in Fig. 4, for a constant energy and for a given monodisperse sample of highly diluted (5%) spheres the saturation point of *μ*_*d*_ as a function of *ξ* occurs at *ξ* = 2*R* and thus directly relates to the feature’s diameter^19^. According to this, the three graphs of Fig. 4 illustrate three distinct regimes of *μ*_*d*_ as a function of *ξ*. The 2 *μm* diameter spheres are within the *μ*_*d*_ saturation regime (*ξ* ≥ 2*R*) as also shown in Fig. 3(d), the 6 *μm* ones are at the *μ*_*d*_ increasing regime (*ξ* < 2*R*) and the 10 *μm* ones near the initial slow *μ*_*d*_ increasing regime (*ξ* < 2*R*). For the case of 2 *μm* diameter spheres or smaller, the point at which *μ*_*d*_ saturates can be reached by our system’s *ξ* range. Exploiting and mapping the points around this saturation regime could be a promising method for extracting size information given certain *a priori* sample knowledge.

## Discussion

In this article, we start from the established quantitative interpretation of dark-field signal originating from uncorrelated spheres and perform both an experimental and analytical quantitative extension of the direct link between XGI dark-field imaging and USAXS, for concentrated monodisperse colloids of varying diameters and volume fractions reaching 40%. This extension enables the quantitative incorporation of a structure factor in the imaging regime, which accounts for interparticle scattering interference and is essential for the successful characterization of concentrated colloids. Based on the results, it is necessary to consider a structure factor if attempting to extract quantitative information from dense or granular biological or material science samples, since otherwise contrast variations can be easily misinterpreted.

In addition, we propose an alternative method of probing a certain range of correlation lengths through energy selectivity, while keeping the XGI optical components and the sample at fixed positions along the optical z-axis. For the appropriate correlation length range which has to span around the *μ*_*d*_ as a function of *ξ* saturation regime, the combination of this method and the appropriate form and structure factor models can potentially result in obtaining quantitative information, such as sphere size or sample density from dark-field images of concentrated granular objects. More importantly, we are currently working to implement this method using a polychromatic X-ray source table-top XGI and an energy-resolving single-photon-counting detector. This should allow mapping a selected correlation length range, during a single phase-stepping acquisition at fixed magnification and field of view, without altering the XGI component positions along the optical z-axis.

## Methods

In XGI, three types of contrast are recovered by analysing the dense interference fringes obtained at the Talbot or Lohman distance downstream the phase grating G1. Since the period of the fringes is much smaller than the pixel size of an X-ray detector, a second absorption or analyzer grating G2 is positioned in front of the detector. Image acquisition requires one of either G1 or G2 to be moved in sub-period traversal steps with respect to the other. The grating motion creates a sinusoidal-like phase-stepping curve and is repeated twice, once to obtain the “object-less” reference curve and a second time for the distorted “object” pattern. Absorption, differential phase and dark-field signals can be then defined as the sinusoid’s reduction in mean intensity, phase shift and dampening of amplitude, respectively.

The evaluation of the proposed framework linking the USAXS and dark-field regimes, as well as the demonstration of the induced effects of *S*(*q*) on dark-field, were performed experimentally at TOMCAT the tomographic imaging beamline of the Swiss Light Source at Paul Scherrer Institute, Villigen. The XGI was implemented at a 3^rd^ Talbot order *π*-shifting arrangement and was operated in projection mode using a Si <111> crystal monochromator, which provided a relative energy bandwidth of 10^−4^. The interferometer’s design energy was 25 *keV*, the pitch of the gratings G1 and G2 was 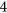 and 2 *μm*, respectively, the inter-grating distance was 12 ± 0.1 *cm* and the samples were placed prior to G1. At the design energy of 25 *keV*, the XGI’s correlation length was equal to *ξ* = 3.0 ± 0.2 *μ*m and the system’s components remained fixed at all times along the optical z-axis. The X-rays were converted to visible light through a 350 *μm* LuAg:Ce scintillator which was then detected using a scientific CMOS pco.edge 4.2 camera with a pixel size of 6.5 *μm*. To perform dark-field imaging, the pixel size has to be significantly larger than the imaged particles thus 10x binning was implemented and the effective pixel size used for all the measurements was 65 *μm*.

The imaged samples consisted of varying in concentration suspensions of monodisperse SiO_2_ microspheres in either glycerin or water, with diameters *d* = 10.0, 7.75, 6.4, 2.0 and 1.86 *μm* and density of approximately *ρ *= 2.0 *g*/*cm*^3^ (according to the manufacturer, Corpuscular Inc. and Cospheric LLC). Dry SiO_2_ microspheres were used to prepare the suspensions by adding the appropriate amount of solute into 5 ml of solvent (glycerin or de-ionized water) at room temperature. Glycerin was used for the fraction of volume control experiments due to its high viscosity (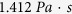
32) with respect to water (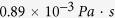
33) at 25 °C, which slowed down the precipitation of the microspheres. The suspensions were vortexed for 5 min and sonicated using an ultrasonic bath for 10-minute cycles until the spheres were fully dispersed. Then, 1 ml of each suspension was transferred into separate 4 mm thick rectangular PMMA cuvettes. The 7.75 and 1.86 *μm* microspheres were used for the fabrication of suspensions with increasing concentrations from approximately 5% up to 40%. These samples were all imaged at 25 *keV* and thus at constant *ξ*, to illustrate the result of incremental fraction of volume increase on dark-field signal.

The 10.0, 6.4 and 2.0 *μm* diameter microspheres were dispersed in water and analyzed a few days after the preparation, in order to ensure they reached the sedimentation state. This set of samples was used to experimentally probe different correlation lengths by means of changing the X-ray energy. This experiment was performed for high concentration (sedimentation) states, where interparticle scattering interference is significant. The main experimental parameters are listed in Table 1.

The fraction of volume *ϕ* was calculated for each sample and at each energy or concentration point, by taking the difference between the absorption values of a cuvette filled with a prepared microsphere suspension and one of pure glycerin or water^18^


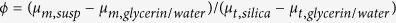


where the numerator is the difference between the measured suspension and glycerin or water linear attenuation coefficients and the denominator the difference between tabulated values taken by the United States National Institute of Standards and Technology XCOM database^34^.

## Additional Information

**How to cite this article**: Gkoumas, S. *et al*. A generalized quantitative interpretation of dark-field contrast for highly concentrated microsphere suspensions. *Sci. Rep.* **6**, 35259; doi: 10.1038/srep35259 (2016).

## Figures and Tables

**Table 1:** Experimental parameters.

	*d* [*μm*]	*ρ* [*g*/*cm*^3^]	*E* [*keV*]	*ξ* [*μm*]	ϕ [%]
Probing *ϕ* experiment	7.75, 1.85	2.0	25	3.0	5, 10, …, 40
Probing 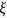 experiment	10.0, 6.4, 2.0	2.0	22, 25, …, 37	3.4, 3.0, …, 2.0	sedimented
